# Risk factors for inadequate bowel preparation and quality improvement strategies in colonoscopy: a narrative review

**DOI:** 10.3389/fsurg.2026.1969199

**Published:** 2026-10-13

**Authors:** Shanshan Ma, Xinyu Zhou

**Affiliations:** Sun Yat-sen University Cancer Center Gansu Hospital (Gansu Provincial Cancer Hospital), Lanzhou, China

**Keywords:** bowel preparation quality, colonoscopy, optimization strategies, patient adherence, purgatives, risk factors

## Abstract

Colonoscopy is the gold-standard screening test for colorectal cancer, yet its diagnostic yield relies heavily on adequate bowel preparation. Despite standard protocols, 20%–30% of procedures still have inadequate cleansing, which lowers adenoma detection, prolongs procedures, increases repeat examinations, and wastes resources. Preparation quality is influenced by multiple interacting factors: patient characteristics (age, comorbidities, medication use, adherence), regimen features (purgative type, dose, split-dose scheduling), and healthcare delivery (education quality, appointment timing). Most previous studies have examined these factors in isolation, leaving a gap in integrated, evidence-based guidance. This narrative review synthesizes current knowledge on determinants of preparation quality, with emphasis on emerging strategies such as risk-stratified protocols, low-volume purgatives, digital health tools (mobile apps, SMS reminders), and AI-based real-time assessment. We aim to provide a practical framework for individualized preparation, thereby improving both the efficiency and quality of colonoscopy-based screening.

## 1 Introduction

Colonoscopy is essential for colorectal cancer (CRC) screening and early detection, but its preventive value depends on the quality of bowel preparation. Adequate preparation allows clear visualization of the mucosa, which is prerequisite for high detection rates of adenomas and reduction of interval cancers (1). Nevertheless, inadequate preparation occurs in one-fifth to one-third of procedures, causing longer procedure times, more failed cecal intubations, lower adenoma detection, added repeat examinations, and considerable cost (2). In China, the rapid expansion of endoscopy has made quality assurance a priority. A national survey covering thousands of hospitals showed that from 2,015 to 2,019, diagnostic colonoscopy volume increased 1.44-fold, and the adenoma detection rate (ADR) rose from 14.9% to 19.3%, though still below Western figures (3). The 2,024 Chinese Digestive Endoscopy Report noted that by 2023, ADR reached 26.9%, but bowel preparation quality—a key driver of ADR—remains a major obstacle (4). Hence, a comprehensive review of risk factors and optimization measures is clinically relevant for improving colonoscopy quality in China and reducing CRC burden.

The determinants of preparation quality are diverse, covering patient attributes, purgative regimens, education, and system processes. Established patient-level risk factors include older age, male sex, obesity, diabetes, chronic constipation, history of poor preparation, inpatient status, and use of opioids or tricyclic antidepressants. These factors probably impair cleansing through slowed motility, altered drug handling, or poor compliance. Regimen-related efficacy also varies substantially. For instance, a large Dutch screening study found that while 4L polyethylene glycol (PEG), 2L PEG-ascorbate, and ≤1L low-volume regimens (e.g., magnesium citrate-based, 1L PEG plus sulfate) all met the European Society of Gastrointestinal Endoscopy (ESGE) minimum standard [Boston Bowel Preparation Scale (BBPS) ≥ 6], the 2L preparation gave a significantly higher adequate-cleansing rate (98.0%) than the 4L (97.1%) and ≤1L (97.0%) options (2). The US Multi-Society Task Force recommends split-dose as preferred, while accepting same-day dosing for afternoon procedures, though it is notably less effective for morning colonoscopies (5).

Regarding patient education, traditional verbal or written instructions are often poorly retained. Enhanced education via mobile apps or SMS reminders consistently improves adherence and preparation quality. System-level factors—such as appointment lead times and routine quality monitoring—also matter. A Dutch registry study reported that continuous quality feedback was associated with a 97.1% adequate preparation rate in screening cohorts (6).

Recent advances in artificial intelligence (AI), mobile health, and novel purgatives have spurred risk-tailored optimization strategies. For AI-assisted detection, a meta-analysis of seven randomized trials found that computer-aided detection (CADe) reduced adenoma miss (risk ratio 0.46) and polyp miss (0.43), with additional benefit for sessile serrated lesions (7). In its 2025 position statement, ESGE gave a weak recommendation for CADe in screening/surveillance, acknowledging modest absolute benefit but patient preference for AI assistance (8). AI has also been applied to polyp size measurement; one study using a water-column reference achieved median error of only 7.4%, versus 25.1% by visual estimation and 20.0% by forceps (9). Low-volume purgatives are increasingly preferred due to better tolerability with comparable efficacy. In the Dutch study, adding bisacodyl to ≤1L ultra-low-volume regimens raised BBPS adequacy (97.3% vs. 95.6%) but increased discomfort (OR 1.47) (2).

In summary, bowel preparation quality is affected by patient, regimen, education, and process factors, and its optimization is a key lever for overall colonoscopy quality. Although many strategies are effective, precise targeting based on individual risk profiles and effective integration of AI and digital tools into routine care remain important research directions. This review systematically synthesizes these determinants and mainstream interventions, aiming to guide clinical practice and quality improvement.

## 2 Determinants of bowel preparation quality

### 2.1 Patient-related factors

Advanced age (>65 years) is an established independent risk factor for poor preparation, attributed to age-related decline in gastrointestinal motility, which reduces propulsive efficiency and purgative excretion (10). Older patients often have impaired swallowing and multiple comorbidities, further compounding risk (11). A prospective study of hospitalized patients ≥60 years reported poor preparation in 33.6%, with chronic constipation as a significant independent predictor (OR 3.307) (12).

Beyond age, comorbidities—including diabetes, chronic constipation, Parkinson's disease, and prior abdominal surgery—can compromise cleansing. Diabetes impairs motility and delays gastric emptying (13); prior surgery may alter anatomy or cause adhesions, affecting purgative distribution (12). Patient adherence to the regimen (purgative intake, dietary restrictions, fluid consumption) is another critical factor; one study found that non-adherence to PEG dosing increased the risk of inadequate preparation 4.576-fold (12). Psychosocial issues—anxiety, depression, low health literacy—also hinder proper execution of instructions. The fact that enhanced education via smartphone apps improves adherence and outcomes (14) indirectly underscores the role of psychological and cognitive factors.

Male sex has been consistently associated with inadequate preparation in adult cohorts; proposed mechanisms include longer colonic transit/segment length, higher prevalence of diverticular disease, and lower adherence to dietary and fluid instructions (5, 12, 15). Opioids and tricyclic antidepressants independently increase risk by delaying colonic transit: opioids activate enteric *μ*-opioid receptors, inhibiting peristalsis and increasing sphincter tone, whereas tricyclics exert anticholinergic effects that reduce propulsive activity and worsen constipation; these effects are compounded by polypharmacy and advanced age (5, 12, 15).

Patient adherence to the regimen (purgative intake, dietary restrictions, fluid consumption) is another critical factor; one study found that non-adherence to PEG dosing increased the risk of inadequate preparation 4.576-fold (16). Psychosocial issues—anxiety, depression, low health literacy—also hinder proper execution of instructions (17–19). The fact that enhanced education via smartphone apps improves adherence and outcomes, indirectly underscores the role of psychological and cognitive factors (20–22). Table 1 summarizes the main patient-related risk factors, their reported effect sizes, and proposed mechanisms.

**Table 1:** Risk factors for inadequate bowel preparation: effect sizes and mechanisms.

Risk factor	Reported effect size/association	Proposed mechanism	References
Age >65 years	Independent risk factor; poor preparation in 33.6% of hospitalized patients ≥60 years; chronic constipation OR 3.307	Age-related decline in gastrointestinal motility, reduced propulsive efficiency and purgative excretion; impaired swallowing and comorbidities	(10–12)
Male sex	Consistently associated with inadequate preparation in adult cohorts; effect size varies by population	Longer colonic transit/segment length; higher prevalence of diverticular disease; lower adherence to dietary/fluid instructions	(5, 12, 15)
Diabetes	Independent risk factor; contributes to inadequate preparation	Impaired motility and delayed gastric emptying	(12, 13, 15)
Chronic constipation	Independent predictor; OR 3.307 in hospitalized elderly	Slow colonic transit, hard stool, incomplete evacuation	(12, 15)
Non-adherence to PEG dosing	OR 4.576 for inadequate preparation	Insufficient purgative intake; poor dietary/fluid compliance	(16)
Opioids	Independently increase risk; no pooled estimate reported	Activate enteric μ-opioid receptors, inhibit peristalsis, increase sphincter tone	(5, 12, 15)
Tricyclic antidepressants (TCA)	Independently increase risk; no pooled estimate reported	Anticholinergic effects reduce propulsive activity and worsen constipation	(5, 12, 15)
Inpatient status	Associated with poorer preparation; split-dose, 1-L PEG, and ≥75% intake were protective in hospitalized patients	Longer fasting/waiting, altered motility, comorbidity, medication use	(15)
Prior poor preparation	Established risk factor in guidelines; no pooled estimate reported	Multifactorial: adherence, motility, regimen intolerance	(5)
IBD/UC	High-risk for inadequate preparation; predictors include male sex, prior ileal/colonic surgery, and stricturing CD (23); dedicated risk assessment recommended; high cumulative procedure burden; no pooled effect size reported (23–32)	Active inflammation, altered motility, prior colorectal surgery, opioids/anticholinergics, anemia, malnutrition, psychological distress	(23–32)

### 2.2 Regimen-related factors

The choice of purgative substantially affects both efficacy and tolerability. PEG is considered the gold standard due to safety and effectiveness, but its 4-L volume often causes nausea and bloating, reducing completion rates (33). Low-volume alternatives, such as sodium picosulfate with magnesium oxide and citric acid (SPMC), are increasingly used. A pediatric study showed SPMC had comparable efficacy to PEG but better tolerability and acceptability (33), and an adult subgroup analysis confirmed its favorable profile across age groups, including those ≥65 years (11). Lactulose-based regimens, largely evaluated in Eastern populations, are non-inferior to PEG for overall cleansing in several RCTs and meta-analyses, with less nausea and bloating but more abdominal cramping and longer preparation-to-colonoscopy intervals; lactulose may therefore be considered for patients who cannot tolerate PEG, although guideline endorsement remains limited (34, 35).

Regarding timing, split-dose administration (dividing the purgative into evening-before and morning-of doses) is guideline-preferred and improves right-colon cleansing (10), though scheduling conflicts may limit implementation. Dietary restrictions remain debated; although a full-liquid diet has been traditional, recent evidence supports a low-residue diet. A randomized trial reported that patients on a low-residue diet achieved higher BBPS scores and better tolerability (less hunger, nausea, bloating) than those on full liquids (36). Adding prokinetics like mosapride or prucalopride can enhance quality without increasing adverse events, particularly in elderly or poorly tolerant patients (10, 37).

### 2.3 Healthcare system–and process-related factors

System factors significantly influence preparation quality. Afternoon colonoscopies are often associated with poorer cleansing than morning ones, likely due to longer fasting and waiting times, which may slow motility or reduce purgative effectiveness. The interval between the second dose and procedure start is also critical—too short leaves fluid, too long allows secretion re-accumulation.

The quality of patient education from staff is another key determinant. Traditional verbal instructions, lacking visual aids, often lead to poor retention. Enhanced education—via telephone, SMS, video, or mobile apps—substantially improves quality. A meta-analysis of 18 trials found that enhanced education increased adequate preparation rates (OR 2.59) (38). Smartphone apps specifically raised BBPS scores and ADR, while improving adherence to low-residue diet and split-dose (14). Similarly, SMS reminders also improve quality and satisfaction (39). A recent multicenter trial confirmed that automated electronic reminders (E-reminder) effectively enhance preparation quality and adherence (40). These findings highlight the importance of systematic, standardized patient education.

## 3 Development and application of risk assessment tools

### 3.1 Comparison of existing risk assessment models

Clinical scoring systems based on variables such as age, medication history, and constipation are commonly used to predict inadequate preparation, with BBPS as the reference. However, their sensitivity and specificity vary across populations. A pediatric study found that age, sex, and obesity were not significant predictors in children, suggesting adult-derived models may not apply (41). Elderly patients, who have higher risk due to physiological decline and comorbidities, also show limited predictive accuracy with existing models (42). Hence, despite simplicity, heterogeneity in performance restricts broad use.

Machine learning models that integrate multidimensional electronic health record (EHR) data—including medical history, labs, and medications—have achieved superior accuracy. One study using deep learning for image classification reported AUC >0.80, outperforming conventional scores (43). Another showed that risk-stratified prioritization for colonoscopy increased advanced neoplasia detection (44). These models handle complex nonlinear relationships, enabling more precise identification of high-risk individuals. However, they depend heavily on robust health information systems, posing challenges in low-resource settings. Paper-based risk tools have been validated as feasible alternatives in such environments (45).

In practice, risk stratification can guide resource allocation—directing enhanced education or stronger purgatives to high-risk patients. One study showed that risk-adapted screening could outperform fecal immunochemical testing while keeping colonoscopy burden manageable (44). But effective implementation requires EHR integration for automated risk calculation and decision support. In settings without such infrastructure, paper-based tools, though effective, are less efficient and accurate (45). Future work should prioritize scalable, high-performance risk tools suitable for diverse healthcare environments.

### 3.2 Value of dynamic assessment and real-time intervention

Dynamic assessment via mobile apps or telephone follow-up during preparation can identify patients who fail key steps and provide corrective guidance in real time, significantly improving quality. A multicenter trial reported that an automated reminder system (calls, SMS, web) achieved higher adequate preparation rates (60.7% vs. 54.5%, *P* = 0.01) (40). Similarly, smartphone-based enhanced education raised BBPS scores and ADR, and improved adherence (14). The strength of dynamic interventions lies in their immediacy and interactivity, allowing personalized guidance beyond static education.

A simple patient-reported indicator—the color of the last bowel movement—has been validated as a practical proxy for preparation quality, useful in remote settings without real-time endoscopic assessment. One study using telephone guidance showed the intervention group had significantly higher BBPS scores (*P* < 0.001) and cecal intubation rates (*P* = 0.027) (46). Such assessments enable providers to detect inadequate preparation before colonoscopy and take remedial actions (regimen adjustment or rescheduling). Mobile-app-collected data can also feed back to refine prediction models.

Dynamic assessment also improves patient experience and adherence. A WeChat-based video education study reported higher BBPS scores (7.02 vs. 6.45, *P* < 0.05) and shorter insertion times in the intervention group (47). This mobile social media approach not only improved adherence but also enhanced communication. Early identification of intolerance to purgatives allows timely strategy changes, reducing adverse events. Integrating dynamic assessment into routine care is a key strategy for optimizing colonoscopy quality, especially in telemedicine and resource-limited settings.

## 4 Optimization interventions: individualization and digitalization

### 4.1 Risk-stratified tailored regimens

Adopting differentiated preparation protocols based on patient risk profiles is a core strategy for optimizing quality and patient experience. For low-risk patients, a standard low-volume PEG regimen with one education session is usually sufficient. Studies show that a 1-day low-residue diet provides comparable cleansing to 2- or 3-day regimens, with better tolerability and satisfaction (48, 49). For high-risk groups—elderly, hospitalized, diabetics—enhanced protocols are needed. In elderly patients (≥65 years), split-dose PEG plus mosapride improved quality and reduced bloating (10). In hospitalized patients, split-dose, 1-L PEG-based purgatives, and intake of ≥75% of the prescribed volume were independent protective factors (15). For those with chronic constipation or diabetes—both independent risk factors (12, 15)——tailoring the purgative and intensifying dietary restrictions (e.g., semi-liquid diet 2 days before) may be more effective.

In special populations such as those with diabetes or renal impairment, careful selection of purgative composition and electrolyte monitoring are crucial. Sodium phosphate should be avoided in diabetic nephropathy due to acute phosphate nephropathy risk. One study comparing PEG with sodium phosphate in unsedated colonoscopy found that although sodium phosphate had better cleansing, PEG resulted in shorter intubation and greater comfort (50). For renal insufficiency, PEG-based regimens are safer. Combination formulations like magnesium sulfate with citrate offer better palatability and acceptance for patients who cannot tolerate large-volume PEG (33). A pediatric study confirmed that SPMC had superior tolerability and acceptability compared with PEG-ELS (33). Overall, tailoring by age, comorbidities, and medications is fundamental.

In patients with inflammatory bowel disease (IBD), especially ulcerative colitis (UC), bowel preparation is uniquely challenging because these patients undergo repeated surveillance colonoscopies and often have active inflammation, altered motility, prior colorectal surgery, chronic opioid/anticholinergic use, anemia, malnutrition, and psychological distress (24, 25). Active UC with diarrhea may paradoxically cause rapid transit and poor retention of purgative, whereas quiescent colitis with chronic constipation or strictures may impair cleansing (26). A dedicated risk assessment should document disease activity, extent, surgical history, medication use, and prior preparation quality (27). Recent evidence supports low-volume 1 L PEG-ASC as an effective and well-tolerated option in IBD/UC. A multicenter observational study of 284 IBD patients (141 UC, 143 CD) reported overall cleansing success of 94.2% with 1 L PEG-ASC (CD 93.9%, UC 94.6%; mean BBPS 7.47 ± 1.44), complete intake in 92.9%, and willingness to repeat in 86.9%, with severe nausea in only 0.3% and no other severe adverse events (28). In 506 IBD outpatients, 1 L PEG-ASC achieved significantly higher adequate preparation than 2 L PEG (89.8% vs. 83.8%, *P* = 0.048; median BBPS 8 vs. 6, *P* < 0.001) and was independently associated with adequate preparation (OR 1.70, 95% CI 1.00–2.90); male sex, prior ileal/colonic surgery, and stricturing CD predicted inadequate preparation (23). A 2025 systematic review and meta-analysis of 10 RCTs (1,479 IBD patients) found high-certainty evidence that low-volume PEG is comparable to high-volume PEG for preparation success (RR 0.98, 95% CI 0.88–1.09) with better acceptability; low-volume non-PEG options also showed similar success (picosulfate RR 0.89, 95% CI 0.78–1.01; sulphate RR 0.98, 95% CI 0.91–1.05) (29). In a multicenter RCT of 311 UC patients, 1 L PEG was non-inferior to 3 L PEG for BBPS (6.80 ± 1.57 vs. 7.24 ± 1.43; non-inferiority *P* = 0.0007), with better tolerance (8.28 ± 1.67 vs. 7.63 ± 2.31, *P* = 0.001), higher willingness to repeat (*P* = 0.014), and less nausea (14.5% vs. 28.0%, *P* = 0.007) (30). A prospective study of 103 IBD patients using split 1 L PEG (500 mL × 2) reported adequate cleansing (BBPS ≥6) in 85.4%, complete intake in 99%, and willingness to repeat in 86.4% (31). In a multicenter observational study of 284 IBD patients, 1 L PEG-ASC achieved overall and right colon cleansing success in 94.2% of cases, with complete intake in 92.9% and willingness to repeat in 86.9% (28). Accordingly, split-dose low-volume PEG, particularly 1 L PEG-ASC, is generally preferred in IBD/UC; sodium phosphate should be avoided in severe colitis or renal impairment (23, 26, 28–32). In patients with active UC, individualized assessment of disease activity, extent, surgical history, medication use, and prior preparation quality remains essential, and risk factors such as male sex, previous intestinal surgery, and stricturing CD should prompt intensified preparation strategies (23). Split-dose low-volume PEG is generally preferred; sodium phosphate should be avoided in severe colitis or renal impairment (26, 32). Enhanced education and early telephone/app-based follow-up are particularly important in IBD because of the high cumulative procedure burden (51, 52).

### 4.2 Efficacy of digital health education tools

Digital tools—short-video platforms and mobile apps—are increasingly replacing paper instructions. A prospective, endoscopist-blinded trial showed that smartphone-based enhanced education yielded significantly higher BBPS scores (7.6 vs. 6.7) and reduced inadequate preparation from 17% to 8%, along with higher ADR (35% vs. 27%) and better adherence to diet and split-dose (14). Multimedia education, through visual and interactive formats, improves comprehension and execution, particularly for patients with lower education levels. One study noted that education level significantly influences split-dose success, with college graduates more likely to achieve adequate preparation (53). Hence, providing easily understandable digital content tailored to diverse educational backgrounds is important.

Interactive SMS reminder systems are another effective digital intervention. By sending timed reminders for medication, fluid intake, and other instructions, they enhance adherence. A meta-analysis showed that split-dose PEG had higher acceptability and less nausea than single-dose in children (54). SMS can simulate split-dose scheduling, helping patients complete intake on time. Virtual reality (VR)-based simulation education is emerging; immersive preoperative experience may reduce anxiety and improve adherence in elderly patients. Computer-based education (CBE) was non-inferior to nurse counselling and reduced patient work absenteeism (55). Overall, digital tools improve efficiency and reduce inter-operator variability through standardized delivery, thereby ensuring broader quality.

### 4.3 Novel purgatives and administration routes

Next-generation low-volume purgatives are a major direction for improving tolerability and completion. The 1-L PEG-3350/ascorbic acid formulation (NER1006) has shown equivalent or superior efficacy to conventional 4-L or 2-L regimens. A prospective multicenter study reported that NER1006 achieved a significantly higher cleansing success rate (90.1%) than 4-L (72.4%) and 2-L (74.1%), with superior high-quality right-colon cleansing (41.4% vs. 15.9% and 12.0%) (56). Meta-analyses confirmed NER1006 outperforms conventional preparations in overall and right-colon cleansing, though with slightly more mild-to-moderate adverse events (57). By reducing required fluid to 1,500–2000mL, this formulation decreases bloating and nausea, enhancing tolerability and completion (56). A US real-world survey found that 89.9% of patients were satisfied or very satisfied with NER1006 (58).

Head-to-head randomized and real-world comparisons between 1-L PEG-ASC and SPMC show broadly comparable adequate preparation rates, but SPMC is often associated with better palatability and acceptability, whereas 1-L PEG-ASC may provide superior right-colon cleansing in some trials (33, 56, 59–61). Lactulose-based regimens, largely evaluated in Eastern populations, are non-inferior to PEG for overall cleansing in several RCTs and meta-analyses, with less nausea and bloating but more abdominal cramping and longer preparation-to-colonoscopy intervals; lactulose may therefore be considered for patients who cannot tolerate PEG, although guideline endorsement remains limited (34, 62, 63).

Oral magnesium sulfate combined with magnesium citrate is another effective low-volume option with improved palatability, suitable for patients unwilling to take large-volume PEG. One study showed SPMC oral solution had consistent efficacy and tolerability across BMI groups (59). A novel sulfate tablet formulation (OST) – 24 tablets with adequate fluid – achieved non-inferior cleansing to PEG-ASC, with good tolerability (64). For salvage after failed preparation, innovative colonic lavage devices (e.g., modified rectal balloon catheters) have shown high efficacy, though not yet widely adopted. For hospitalized patients or those unable to tolerate oral purgatives, localized lavage may be an effective alternative (15). These developments are driving colonoscopy toward greater efficiency, comfort, and safety. Table 2 summarizes the efficacy, tolerability, and practical considerations of commonly used and emerging purgative regimens.

**Table 2:** Purgative regimens: efficacy, tolerability, and practical considerations.

Regimen	Volume	Adequate preparation/BBPS	Tolerability	Notes
4-L PEG	4 L	Adequate cleansing 97.1% in Dutch screening (2)	Nausea, bloating, lower completion rates (33)	Gold standard for safety/effectiveness; high volume limits tolerability (2, 33)
2-L PEG-ASC	2 L	Adequate cleansing 98.0% (2)	Generally better tolerated than 4-L PEG	Met ESGE minimum standard (BBPS ≥6); significantly higher adequate cleansing than 4-L and ≤1-L (2)
1-L PEG-ASC/NER1006	1 L (total fluid 1,500–2,000mL)	Cleansing success 90.1% vs 4-L 72.4% and 2-L 74.1%; high-quality right-colon cleansing 41.4% vs 15.9% and 12.0% (56)	Mild-to-moderate adverse events slightly more than conventional in meta-analysis; less bloating/nausea due to lower volume (56, 57)	Meta-analyses confirm superior overall and right-colon cleansing (57); US real-world survey: 89.9% satisfied/very satisfied (58)
1-L PEG-ASC in IBD/UC	1 L, split-dose; total fluid 1,500–2,000mL	IBD overall cleansing success 94.2% (28); 1 L PEG-ASC vs 2 L PEG: 89.8% vs 83.8%, median BBPS 8 vs 6 (23); UC: 1 L vs 3 L PEG BBPS 6.80 ± 1.57 vs 7.24 ± 1.43, non-inferior (30); split 1 L PEG: BBPS ≥6 in 85.4% (31); meta-analysis low- vs high-volume PEG: RR 0.98 (29)	Better tolerated than higher-volume PEG; less nausea; higher willingness to repeat (28, 30, 31); severe nausea only 0.3% (28)	Split-dose low-volume PEG, especially 1 L PEG-ASC, is generally preferred in IBD/UC; avoid sodium phosphate in severe colitis or renal impairment (23, 26, 28–32)
SPMC	Low-volume (product-dependent)	Comparable efficacy to PEG in pediatric study; favorable profile across age groups including ≥65 years; consistent efficacy/tolerability across BMI groups (11, 33, 59)	Better palatability and acceptability than PEG; suitable for patients unable to tolerate large-volume PEG (33, 59)	Often compared with 1-L PEG-ASC; broadly comparable adequate preparation rates, but 1-L PEG-ASC may provide superior right-colon cleansing in some trials (33, 56, 59)；head-to-head comparisons also include (60, 61)
Lactulose	Low-volume (regimen-dependent)	Non-inferior to PEG for overall cleansing in several RCTs/meta-analyses, largely Eastern populations (34)	Less nausea and bloating; more abdominal cramping; longer preparation-to-colonoscopy intervals (34)	Consider for patients who cannot tolerate PEG; guideline endorsement remains limited (34); see also (62, 63)
Sulfate tablets (OST)	24 tablets with adequate fluid	Non-inferior cleansing to PEG-ASC (64)	Good tolerability (64)	Novel tablet formulation; avoids large-volume liquid (64)

## 5 Quality assurance and artificial intelligence–assisted assessment

### 5.1 Implementation of standardized endoscopy reporting systems

Standardized reporting systems integrate bowel preparation quality assessment into routine practice, providing a unified, quantifiable documentation framework that underpins quality control and improvement. The BBPS is the most widely used tool; its standardized recording minimizes subjective variability and enables comparability across endoscopists and institutions (65). Structured databases incorporating BBPS scores allow benchmarking across facilities, identification of poorly performing segments or physicians, and targeted remedial actions. A study showed that a web-based bowel monitoring system improved BBPS scores and adherence, supporting data-driven feedback (66). Regular staff training on preparation protocols, coupled with performance metrics, helps mitigate variability. A multicenter trial found that automated electronic reminders significantly increased adequate preparation rates (60.7% vs. 54.5%), highlighting the value of standardized workflows (40). Thus, standardized reporting is not merely documentation but a bridge between practice, assessment, and continuous improvement.

### 5.2 Artificial intelligence–based image recognition technology

AI, particularly deep convolutional neural networks (CNNs), offers objective, real-time assessment of bowel preparation quality. Manual BBPS scoring is subjective and variable; AI models, trained on large image datasets, can achieve highly consistent automated scoring. Studies show deep learning-based systems can reach >94% accuracy in assessing adequacy, with concordance with experts exceeding 90%, thus reducing human error (67, 68). These systems can provide intra-procedural alerts about preparation status of specific segments—e.g., detecting residual debris in the right colon—prompting targeted irrigation, potentially increasing ADR by ∼10% (69, 70). Beyond intra-procedural use, AI can analyze withdrawal videos post-procedure to objectively evaluate intervention effectiveness (e.g., different purgatives), informing clinical decisions (71). A prospective study confirmed a significant negative correlation between automated BBPS (e-BBPS) and ADR, and proposed a more objective adequacy threshold, laying groundwork for standardized AI application in quality assurance (72).

## 6 Conclusions

The pursuit of better bowel preparation quality epitomizes medicine's ongoing shift from experience-based to data-driven practice. In daily practice, this shift confronts three recurring dilemmas: how to reconcile patient diversity with fixed protocols, how to balance thorough cleansing against patient comfort, and how to move from subjective grading to objective measurement. These dilemmas are not separate—they feed into one another, and their interplay is what ultimately decides whether a preparation succeeds or fails.

In everyday clinical practice, advanced age, chronic constipation, and poor adherence stand out as the risk factors that matter most. What this tells us is that bowel preparation is not a one-time technical task; it is a patient-centred process that begins long before the purgative is taken and ends only when the colonoscope is withdrawn. Risk stratification is meant to direct limited resources toward those who need them most, thereby avoiding the bluntness of universal protocols. Yet we must be honest: most current risk scores come from retrospective datasets or small cohorts, and their performance in real-world settings often falls short. The way forward lies in combining electronic health records, medication history, and lifestyle data through nonlinear machine-learning models that can be updated as new information accumulates—making risk scores truly dynamic rather than static. Digital tools can help patients navigate complex regimens by breaking them into manageable, trackable steps, but their success hinges on patient digital skills and the integration of health information systems, both of which vary widely. Take low-volume or split-dose preparations: they are increasingly preferred because they maintain effectiveness while minimising discomfort, especially for older or frailer patients. Yet direct comparisons between different formulations are few, and the higher price of newer agents may keep them out of primary care. We need pragmatic, multi-setting studies that assess not just efficacy but also real-world cost-effectiveness—otherwise, what works in trials may remain out of reach in practice.

AI-assisted assessment shifts the locus of quality control from retrospective review to instantaneous oversight. The appeal is clear: it supplies objective, quantifiable feedback that can temper the variability inherent in human scoring. But we should not overlook the limitations. Most AI systems to date have been trained on data from single centres, and whether their performance travels well to other settings remains an open question. More importantly, we still lack convincing evidence that these tools materially improve hard clinical endpoints—polyp detection, for instance, or patient outcomes over time. Looking ahead, I see three priority areas. One is to design a truly integrated platform that links electronic health records, mobile applications, AI-generated assessments, and patient behavioural data into a responsive “prediction–intervention–feedback” loop. Another is to mount multicentre, prospective studies that can measure long-term reductions in adenoma miss rates, repeat procedures, and overall costs—not just surrogate indicators. A third is to keep cost-effectiveness and equity firmly in view, so that technological advances do not simply serve well-resourced centres while leaving others behind. The ultimate test, however, is whether these strategies can be made to work in ordinary practice. Until they are, we remain in the realm of expert opinion, not clinical reality.
